# Widespread imprinting of transposable elements and variable genes in the maize endosperm

**DOI:** 10.1371/journal.pgen.1009491

**Published:** 2021-04-08

**Authors:** Sarah N. Anderson, Peng Zhou, Kaitlin Higgins, Yaniv Brandvain, Nathan M. Springer

**Affiliations:** 1 Department of Genetics, Development, and Cell Biology; Iowa State University; Ames, Iowa, United States of America; 2 Department of Plant and Microbial Biology; University of Minnesota; St. Paul, Minnesota, United States of America; The University of North Carolina at Chapel Hill, UNITED STATES

## Abstract

Fertilization and seed development is a critical time in the plant life cycle, and coordinated development of the embryo and endosperm are required to produce a viable seed. In the endosperm, some genes show imprinted expression where transcripts are derived primarily from one parental genome. Imprinted gene expression has been observed across many flowering plant species, though only a small proportion of genes are imprinted. Understanding how imprinted expression arises has been complicated by the reliance on single nucleotide polymorphisms between alleles to enable testing for imprinting. Here, we develop a method to use whole genome assemblies of multiple genotypes to assess for imprinting of both shared and variable portions of the genome using data from reciprocal crosses. This reveals widespread maternal expression of genes and transposable elements with presence-absence variation within maize and across species. Most maternally expressed features are expressed primarily in the endosperm, suggesting that maternal de-repression in the central cell facilitates expression. Furthermore, maternally expressed TEs are enriched for maternal expression of the nearest gene, and read alignments over maternal TE-gene pairs indicate that these are fused rather than independent transcripts.

## Author summary

While parents contribute equally to the DNA of offspring, some genes are imprinted, or differentially expressed depending on which parent the allele is passed from. In plants, imprinted genes are expressed in the endosperm, and prior studies have missed many imprinted genes. Here, we present a new method which identifies imprinted genes and transposable elements using all parental differences in sequence, including presence-absence variation between parents, rather than single nucleotide polymorphisms alone. We find that the majority of imprinted genes with maternal expression are genes and transposable elements that are present in only one genome and that are not shared with other grass species. These transcripts are often expressed primarily in the endosperm where epigenetic marks on the maternal alleles are distinct from the rest of the plant. We also find evidence of fused transcripts with transposable elements upstream of genes, implicating common regulation for imprinted transposable elements and some nearby genes.

## Introduction

Imprinted genes showing parent-of-origin based patterns of expression were first identified in maize [1] and have since been identified in a variety of flowering plants [2–6]. In plants, imprinted expression is primarily observed in the endosperm, which is a nutritive tissue of the seed that is formed when the diploid central cell is fertilized by one of the two sperm cells delivered by the pollen tube. The central cell is epigenetically distinct from most vegetative cells in the plant due to DNA demethylation targeted primarily to Transposable Elements (TEs) [7–9]. This demethylation acts as a primary imprint that distinguishes the female and the male alleles in the endosperm. Maternal and paternal alleles are further distinguished through differential accumulation of histone modifications such as H3K27me3 [10,11] which often marks the maternal allele of paternally expressed genes (PEGs) while maternally expressed genes (MEGs) often show differences in DNA methylation alone [12].

Imprinting has been studied at the genomic level in many plant species [2–6]. While some important genes have conserved imprinting in many species [13], several studies have observed variable imprinting for other genes, with inconsistent imprinting within genotypes of a single species or across species [14,15]. However, understanding the rate of turnover and the origins of imprinted expression patterns has been challenging due in part to methodological inconsistencies across studies and the limitations of available SNPs for allele calls. In Arabidopsis, applying consistent methods and cutoffs across studies reduces apparent variability in imprinting calls [16,17], however many genes cannot be assessed due to a lack of informative SNPs. A lack of SNPs can be due to identical sequence or unalignable regions resulting from large structural changes or presence-absence variation (PAV) of whole genes or features. In maize, many genes and TEs exhibit PAV among genotypes [18–20]. This limits the ability to use SNP-based allele-specific expression analyses to study imprinting, especially for transposons and variable genes. In this study, we develop an alternative approach that relies upon comparisons of expression in reciprocal crosses to assess the imprinting of both conserved and variable genes and TEs across maize genotypes with whole genome assemblies, revealing imprinting for many transposable elements and variable genic sequences.

## Results and discussion

**Fig 1:**
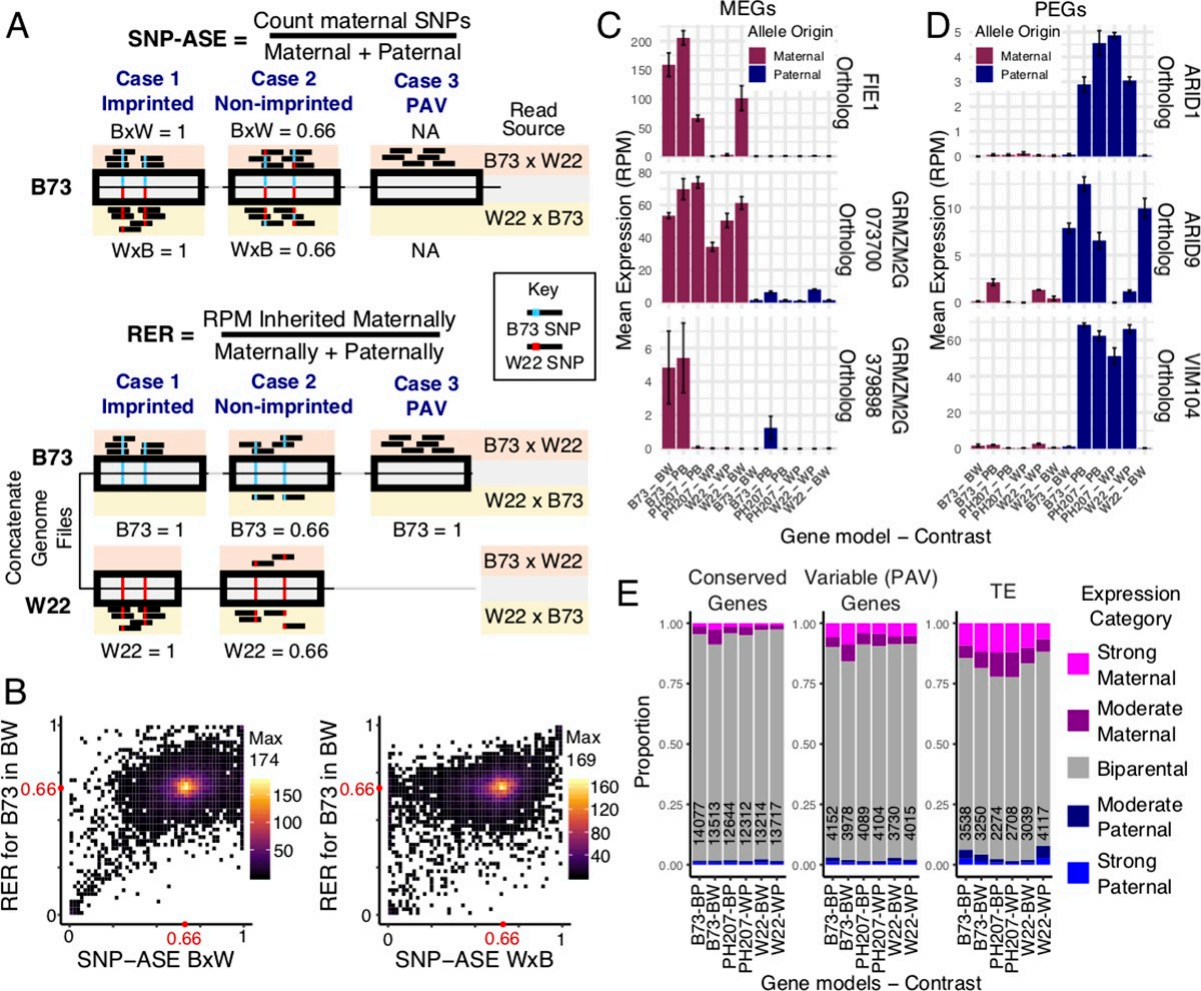
Assessing imprinted expression pattern in maize. A) The method for defining imprinting using SNP-ASE versus RER illustrating an example of an imprinted gene distinguishable by SNPs (Case 1), a non-imprinted gene (Case 2), and an imprinted PAV gene (Case 3). In SNP-ASE, reads mapping to a SNP-corrected reference genome are assigned to alleles based on the SNP supported. In RER, reads are assigned to a concatenated reference genome and retained at unique positions. Both methods can be used to assess imprinting for shared genes distinguished by SNPs, but only RER can assess imprinting for PAV features. B) Comparison of SNP-ASE and RER for B73 alleles assessable with at least 10 unique (RER) and informative (SNP-ASE) reads in the B73 x W22 crosses. The values plotted showi the averages across three biological replicates. SNP-ASE is calculated separately for the B73 x W22 cross (left) and reciprocal W22 x B73 cross (right), while RER is calculated with information from both reciprocals and plotted as the y-axis in both plots. The heat represents the number of genes in each pixel of the plot, and red dots indicate the expected ratios for non-imprinted expressed genes. C-D) Expression patterns of known MEGs (C) and PEGs (D) in this dataset using mapping from concatenated genomes. X-axis shows the source of the gene model and the genome contrast (where B = B73, W = W22, and P = PH207), and bars are colored based on whether the allele plotted is inherited maternally (maroon) or paternally (navy). The y-axis shows the mean expression calculated from unique mapping reads using the mapping strategy used for RER. RER values are calculated by dividing the expression when inherited maternally by the sum of expression from both directions of the cross. Error bars represent standard error, and gene IDs for the orthologs in each genome are listed in S2 Table. E) The distribution of RER values for different features across contrasts. RER cutoffs for strong maternal and strong paternal are > 0.9 and < 0.1, respectively, and cutoffs for moderate maternal and paternal are > 0.8 or < 0.2, respectively The total number of features across all expression categories are shown on each bar.

Reciprocal crosses for every pairwise contrast between three maize genotypes with whole genome assemblies (B73 [21], W22 [22], and PH207 [23]) were performed, and 14 days after pollination, endosperm was isolated in triplicate for RNA-sequencing (S1 Table). Two approaches were applied to identify imprinted expression (Fig 1A). The traditional approach for calling imprinting uses Single Nucleotide Polymorphisms to call Allele Specific Expression (SNP-ASE) followed by comparison of biases across reciprocal crosses (methods). The SNP-ASE ratio is calculated by assigning SNP-containing reads to one allele and determining the proportion of informative reads from each allele, providing an estimate of the expression of two alleles within a single sample. This approach can be applied to a single sample and the assessment of imprinting is typically based on consistent bias for the SNP-ASE value in both reciprocal crosses. We developed and implemented an alternative approach where reads are aligned to concatenated genome files composed of the two parental genotypes and the Reciprocal Expression Ratio (RER) was calculated to describe the ratio of expression for features in each genome when inherited maternally versus paternally. Unlike SNP-ASE, the RER is a comparison of expression of a feature in reciprocal crosses and cannot be calculated for a single sample. Calculations of RER rely on the ~15% of reads that map uniquely to a single location in the concatenated genomes (S1 Table). While many reads map equally well to both genomes and are therefore discarded, unique mapping reads are only found in places of the genome with variants distinguishing the alleles (SNPs or indels) or in regions unique to one genome. After assigning unique reads to features including genes and TEs using HTseq, RER was calculated by dividing the expression level (RPM) when inherited maternally by the sum of expression when maternally or paternally inherited. Given that endosperm is composed of two copies of the maternal genome and one copy of the paternal genome, the null expectation for a transcript’s expression is that it will be twice as highly expressed when inherited from the maternal parent compared to the paternal parent. For both SNP-ASE and RER, the average value representing a biparentally expressed gene is 0.67, allowing direct comparison of the methods. A comparison of SNP-ASE and RER reveals general agreement between these two approaches for genes that could be analyzed with SNPs, with the majority of genes expressed at the ratio expected by 2:1 dosage (Fig 1B). Many of the genes showing disagreement between methods in Fig 1B result from genotype-biased expression which exhibits a strong bias in SNP-ASE for a single sample but doesn’t result in bias for RER (S1 Fig). Inaccuracies in SNP-ASE can also arise from erroneous mapping of paralogs when mapping to a single reference genome. To further assess accuracy of RER, expression patterns for three MEGs and three PEGs with conserved imprinting status in maize, rice, and Arabidopsis [4] were assessed (Fig 1C, S2 Table). In most cases with informative reads, clear parental bias in the expected direction was observed for all genes (Fig 1C).

While both methods can be used to define imprinting for shared genes distinguishable by SNPs, only the RER method can capture imprinting for portions of the genome that exhibit PAV. This provides new opportunities to study parent-of-origin biased gene expression for TEs and variable genes. The distribution of RER values was assessed across contrasts for different feature types (S2A Fig), and the proportion of each set that showed parentally-biased expression was summarized based on RER (Figs 1E, S2B and S2C). This revealed that across all contrasts, genes conserved within maize rarely exhibit parent-of-origin biased expression (Figs 1E and S2). On average, < 3% of expressed genes that are present in all three maize genotypes in this study show a strong parental bias (Fig 1E). For genes that exhibit presence/absence variation among maize lines, a higher proportion (> 6%) of expressed genes show high parental bias, with this set representing genes that are accessible using RER but not SNP-ASE. Strikingly, > 11% of expressed TEs show a strong parental bias, with the majority of strongly biased TEs expressed maternally (Fig 1E).

**Fig 2:**
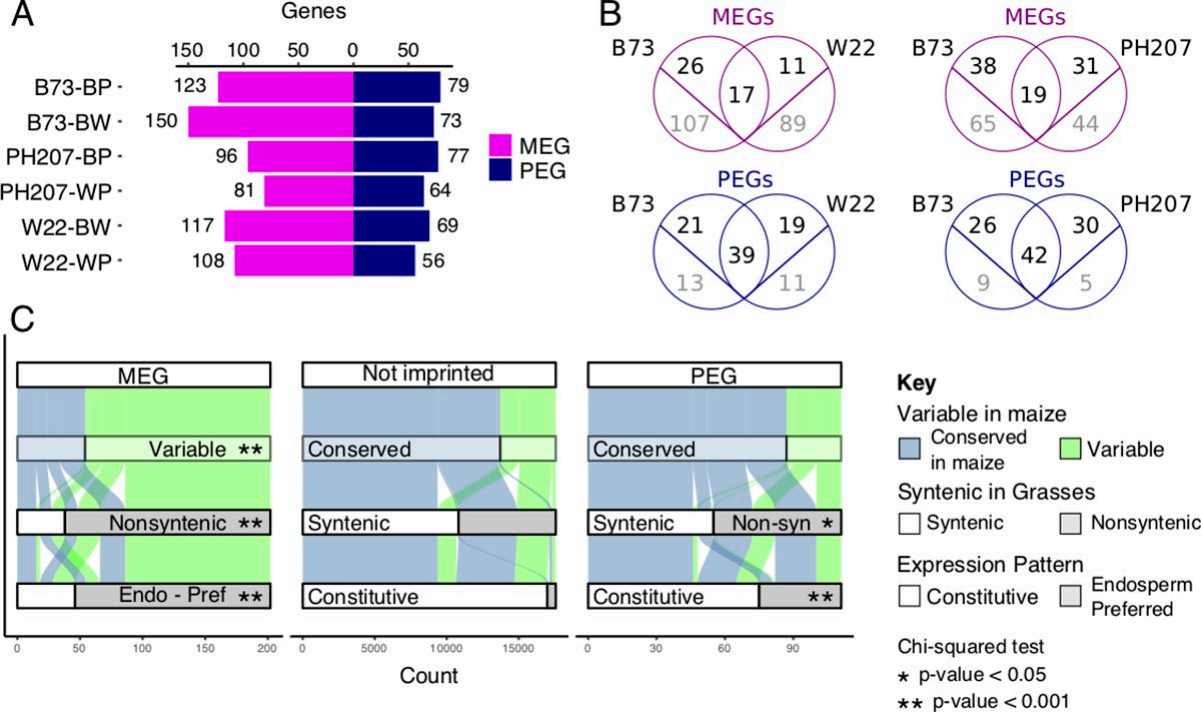
Imprinting of genes defined by RER. A) The number of imprinted genes identified across contrasts using the RER method (see Materials and Methods). MEGs are shown in magenta and PEGs are shown in blue. B) The overlap between imprinted genes across pairwise contrasts. Genes that are shared between genotypes that could be assessed for imprinting are shown in black above the line while imprinted genes unique to one genome are shown in gray below the line. C) Comparison of features for MEGs, PEGs, and non-imprinted B73 genes. Genes are defined as conserved when they are shared with all genotypes present in this study, syntenic when a syntenic ortholog exists in sorghum, rice, foxtail millet, and brachypodium, and endosperm-preferred if expression is primarily restricted to the endosperm (S5 Fig). Asterisks denote significance relative to the Not Imprinted set (chi-squared test).

In order to identify imprinted transcripts, we applied the lfcThreshold option within DESeq2 to test for significance (adjusted p-value < 0.05) over the expected 2:1 gene dosage across reciprocals using three biological replicates. To increase the stringency of imprinting calls, significant hits were further filtered by RER values. Maternally Expressed Genes (MEGs) and Maternally Expressed TEs (matTEs) were filtered for RER > 0.9, while Paternally Expressed Genes (PEGs) were filtered for RER < 0.1. It can be difficult to remove all maternal tissues when isolating endosperm tissue and therefore it is important to limit potential false-positive calls of maternal expression that may result from genes expressed in the maternal seed coat [24]. Previously published RNA-seq data [25] was used to filter out genes whose maternal expression could result from seed coat contamination rather than maternal expression in the endosperm. Pericarp-preferred genes were defined where the mean expression in pericarp was >2-fold higher than the expression in endosperm (S3 Fig). After implementing these criteria and filters, we identified an average of 182 total imprinted genes across all hybrid combinations, with an average of 112 MEGs and 70 PEGs in each (Fig 2A, S1–S3 Data).

**Fig 3:**
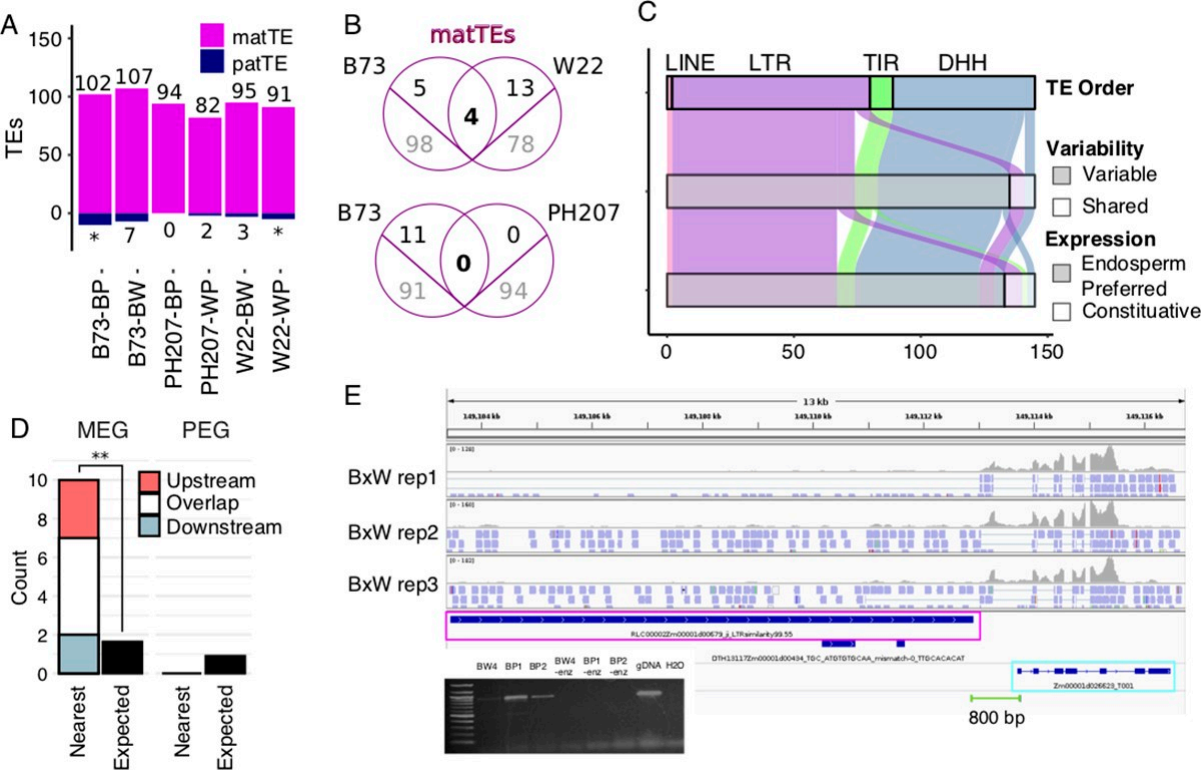
Imprinted TEs defined by RER. A) The number of imprinted TEs across contrasts. matTEs are marked in magenta and paternally expressed TEs are marked in navy. Asterisks denote contrasts where paternally expressed TEs could not be defined (S2 Fig). B) The overlap between matTEs across pairwise contrasts. TEs that are shared between genotypes that could be assessed for imprinting are shown in black above the line while imprinted TEs unique to one genome are shown in gray below the line. C. Features of matTEs in B73. TE orders are abbreviated: DHH = Helitron, TIR = terminal inverted repeat transposon, LTR = LTR retrotransposon, and LINE = long interspersed nuclear element. TE variability is defined by prior work (Anderson et al. 2019). Endosperm-preferred expression is described by patterns across development (S5 Fig). D) Imprinting status of closest gene to matTEs. Expected number is based on the number of MEGs and PEGs that were assessed for imprinting. ** p-value < 0.001 (binomial test) E) IGV view showing a representative example of reads aligning to a matTE near a MEG. Reads are colored by the strand of alignments, where blue = forward strand. Green line shows primer positions for RT-PCR, with positive result shown in gel image.

Since RER considers alleles from each genotype independently, comparative genomic approaches were used to compare the consistency of imprinting for genes discovered in each genome. A comparison of imprinted features in the B73 x W22 reciprocal hybrid endosperm tissue identifies 17 MEGs, 39 PEGs, and 4 matTEs that were consistently imprinted in both genomes (Figs 2B and 3B). A subset of the genes that do not exhibit consistent imprinting are shared between the two genomes. For example, there are 26 MEGs observed only in B73 and 11 only observed in W22 despite the fact that both genomes retain a syntenic ortholog for these genes. For the majority of these shared genes with variable imprinting, the lack of overlap is due to cutoff stringency or lack of coverage rather than true gain or loss of imprinting (S4 Fig). There are many additional cases where imprinted genes are only present in one genome. For PEGs, variable genes represent the minority of non-conserved imprinted genes, with only 13 of 34 B73 PEGs that are not imprinted in W22 variable across genomes. In contrast, for the majority of MEGs with inconsistent imprinting (i.e. 107 of 133 B73 genes in the B73 by W22 contrast), the genes themselves are absent from the other genome. Similar patterns are observed for the B73 by PH207 contrast, though a higher proportion of genes are shared in this contrast. The large number of maternally expressed transcripts with variability in maize suggests that imprinting of non-conserved elements may be far more prevalent than previously detected due to the limitations of SNP-based allele calls.

To understand additional features of imprinted genes, we focused on the B73 genes that were called imprinted in at least one contrast, which included 202 MEGs and 111 PEGs. B73 was selected as the central genotype because it has substantially more expression datasets, syntenic gene information, and functional gene annotations than other genomes. For the genes identified as imprinted, we compared several characteristics relative to genes that were expressed but were not classified as imprinted (S4 Data). First, genes were assessed for variability across maize inbred lines by defining conserved genes as those with syntenic orthologs in B73, W22, and PH207 and variable genes as those without a corresponding gene in at least one genome [26]. This revealed a clear enrichment for variable genes among MEGs (p-value < 0.001, chisq test), but not PEGs, compared to genes that are not imprinted but have enough unique reads to be assessed for imprinting (Fig 2C). We then expanded our evolutionary distance and assessed how many genes in each set are syntenic with other grasses as defined by having a syntenic ortholog in sorghum, rice, foxtail millet, and brachypodium. For genes without imprinting, the majority (62%) are syntenic with other grasses. However, MEGs are highly depleted for syntenic genes (19%) and PEGs show a minor depletion (50%, p-value < 0.05, chisq test). Next, the expression pattern across B73 development was assessed using published RNA-seq data [25]. Since imprinting can arise from either silencing of one parental allele specifically in the endosperm or de-repression of one parental allele in the endosperm, the pattern of expression across tissues was defined as either constitutive or endosperm-preferred (see Materials and Methods, S4 Fig). While only 3% of non-imprinted genes are expressed preferentially in the endosperm, 77% of MEGs and 32% of PEGs show this expression pattern (Fig 2C, p-value < 0.001, chisq test). Many of the MEGs (38%) have no assigned GO term, a 2.8-fold enrichment compared to genes that are not imprinted (p-value < 0.001, chisq test). Since TEs are a common source of new genes and a driver of gene content variation among maize lines, we intersected our imprinted genes with annotated TEs, identifying 26 MEGs and 1 PEG completely within an annotated transposable element. While MEGs and PEGs are annotated as genes in the B73v4 annotation, transcription of a locus does not imply the production of a functional gene product and many “genes” annotated in B73 have TE origins. Evolutionarily conserved genes with synteny to other grasses may be the best candidates for functional genes capable of conferring phenotypes [27], there are certainly some examples of non-conserved genes that can be important for functions such as disease resistance [28].

To further investigate the imprinting of TEs themselves, the RER method was used to define imprinted TEs, with an average of 95 matTEs identified across contrasts (Fig 3A). There are a very small number of paternally expressed TEs, however these were excluded from further analyses due to the low number detected and potential technical complications (Figs 3A and S2). Consistent with the large amount of TE variability among genotypes, the majority of imprinted TEs were unique to one genome (Fig 3B). There are 145 maternally expressed TEs in B73 relative to at least one other genotype, including 72 LTR retrotransposons, 52 Helitrons, 9 TIR transposons, and 2 LINEs (Fig 3C). The vast majority of these TEs (93%) represent specific TE insertions that are polymorphic among the three maize genotypes [20]. Given the high tissue-specificity of TE expression observed previously [29], the tissue-specific expression patterns for matTEs were also assessed. We found that 92% of matTEs are expressed preferentially in the endosperm, suggesting that imprinting is established through de-repression of the maternal allele preferentially in the endosperm and that this is the only stage of development for expression of these elements (Figs 3C and S5). We also assessed some features of these matTEs, finding that matTEs are closer to the nearest gene than non-imprinted genes (p.value < 0.0001, t-test), with a mean distance of 15kb for matTEs vs. 35 kb for non-imprinted genes. For LTR retrotransposons, we are able to confidently assess TE age using LTR similarity as a proxy, where younger elements have more similar LTRs. We find that maternal LTRs are on average older than non-imprinted LTRs (p.value < 0.0001, t-test), with an average of 93% and 95% similarity, respectively.

Since TE families have the potential for coordinated expression responses among members, the families for matTEs were assessed. matTEs are in 84 families, with only one Helitron family containing more than 5 imprinted elements. This family, DHH00002 (DHH2), contains 44 maternally expressed members and is the only Helitron family in B73 that is predicted to have autonomous members. Since prior work has suggested that Helitrons are responsible for creating imprinting by moving PHE1 binding sites around the genome [30], the proportion of DHH2 Helitrons with predicted PHE1 motifs was assessed (S6 Fig). We found that matTEs of this family more frequently have a binding site than elements that are not detected in our analysis (p-value < 0.001, chi square test). However, the proportion with binding sites is similar for imprinted and non-imprinted expressed family members (p.value = 0.38, chi square test), so it is unlikely that PHE1 sites alone are sufficient to confer imprinting of DHH2 Helitrons.

TEs have been proposed as a source of imprinted gene expression [7,31] and coordinate expression of genes and TEs has been observed in other reproductive tissues [32], so we investigated the relationship between imprinted genes and nearby TEs. For every matTE in B73, the closest gene was identified and assessed for imprinting. For 13% of matTEs, the nearest gene is a MEG, which is a significant enrichment (p-value < 0.001, binomial test) and 11.6 times more common than expected based on the proportion of expressed genes that are called MEGs (Fig 3D). In contrast, there were no identified examples of matTEs where the closest gene is a PEG. There were 19 matTEs where the closest gene is one of 15 MEGs (S3 Table). Since TE and gene annotations can overlap and some annotated genes code for TE proteins, we carefully inspected these loci to understand the relationship between these annotated features. In all but one case, the genes overlap (N = 7) or are downstream of the nearest TE (N = 7). In addition to the seven genes overlapping TEs, two downstream genes (Zm00001d046395 and Zm00001d041755) have helicase domains consistent with helitron origin. None of the 15 genes have syntenic orthologs in another grass species, and most (N = 13) exhibit variable presence within maize genotypes. To understand the nature of transcripts, read alignments for matTE-MEG pairs were visualized with IGV. In all cases, reads aligning to both the matTE and corresponding MEG mapped to the same strand without clear separation in read alignments, suggesting that many of these clusters may actually represent single transcripts overlapping multiple features (Figs 3E and S7) rather than clusters of independently imprinted transcripts [33]. To test this experimentally, we performed RT-PCR using primers aligning to the gene and TE, confirming fused transcripts for 4 of 5 loci tested (Figs 3E and S7). Combined with the stranded RNA-seq confirming that the TEs are upstream of the genes within the transcripts, this suggests that some imprinted promoters can drive expression of transcripts overlapping features annotated as TEs and genes.

In summary, we developed the RER method to use information from shared and variable portions of maize whole genome assemblies to identify imprinted expression of genes and TEs in maize. This revealed imprinting of many genes that were undetectable by traditional methods that rely on diagnostic SNPs between parental alleles. The majority of maternally expressed features (genes and TEs) represent young portions of the genome that are variable within maize and non-syntenic with other grasses. It is worth noting that while matTEs are not necessarily younger than non-imprinted TEs, all TE insertions accumulated after the divergence of maize from other grasses. We also observe strong enrichment for MEGs near maternally expressed TEs, suggesting a connection between imprinted TEs and transcripts containing open reading frames. In particular, this may suggest that polymorphic TE insertions can generate novel imprinted expression patterns for nearby sequences, highlighting a potential mechanism for the birth of imprinting. Notably this could occur for an existing gene or could result in imprinting of a novel transposed sequence resulting in a novel “gene.” The observation that many MEGs and matTEs are sequences that have variable presence in different maize genomes is interesting. Imprinting has been proposed to be a phenomena intended to silence TEs in the germline, and maternal expression of TEs and TE-like genes is perhaps an unsurprising outcome of this study. While it is tempting to speculate that the variability in sequence content itself could imply a mechanistic tie between variable sequence and imprinted expression, further studies would be required to better understand the nature of these transcripts and potential cross-talk between parental genomes to create imprinting in a subset of cases.

We previously hypothesized that TE demethylation could result in either MEGs or PEGs [34], however our results suggest that the majority of imprinted TEs are MEGs. The mechanistic bias for this enrichment of MEG TEs is unclear. We speculate that this could arise as a consequence of many PEGs requiring H3K27me3 to silence the demethylated maternal alleles [11,12,35]. In general, H3K27me3 is rarely found over TEs [10,36] and most TEs may lack the sequences necessary for recruitment of H3K27me3 even when they lose DNA methylation. In mammals, imprinting in the placenta has been proposed to result from different defense mechanisms used by male and female germlines to reduce retrovirus proliferation in the germ line [37], and turnover of imprinting could have similar host defense explanations in plant endosperm and animal development [38]. In plants, there are genes with conserved imprinting across plant species that support theories of parental conflict [39] or dosage [40], however the majority of imprinted loci are variable within and across species. By studying imprinting using whole genome assemblies, we are able to better understand the imprinted expression of both shared and variable portions of the maize genome.

## Materials and methods

### Materials

Three maize inbred lines, B73, W22, and PH207, were grown in the field in Saint Paul, MN in the summer of 2018. These genotypes are available through the US National Germplasm System (GRIN). Reciprocal crosses between each pair of genotypes were performed. Ears were collected 14 days after pollination and endosperm was isolated using manual dissection, with approximately 10 kernels per ear pooled for each biological replicate. Paired-end, stranded RNA-seq libraries were created using the Illumina TruSeq Stranded mRNA kit and PE125 sequencing was performed on the Illumina HiSeq 2500 at the University of Minnesota Genomics Center. On average, > 45 million reads were generated per library (S1 Table).

### Sequence alignments for RER

Concatenated genome files were created for each pairwise contrast of parental genomes and assemblies used included B73v4 [21], W22 [22], and PH207 [23]. When necessary, chromosome designations were altered to ensure non-redundant sequence names across parents. Hisat2 index files were created using genome sequences only for each contrast. Gene annotations and disjoined filteredTE annotations available at https://github.com/SNAnderson/maizeTE_variation were combined by first subtracting exon regions from the TE annotations and then combining full gene and TE annotations for each genome. This resulted in a file where reads aligned to exon regions of gene annotations are assigned to genes while reads aligned to TE-containing introns are assigned to TEs. Ambiguous reads are not counted to either feature. Concatenated annotation files were then created for each pairwise contrast using the same chromosomal designation as for the genome files. RNA-seq reads were trimmed using cutadapt [41] and aligned to the concatenated genomes corresponding to the parents using hisat2, using default parameters [42]. Unique-mapping reads to the concatenated genome files were then assigned to features (genes and TEs) using HTseq [43]. Counts to each feature were normalized as reads per million using library size estimates derived from the SNP-ASE method (described below). RER for each annotation (gene and TE) was calculated by dividing the mean expression when inherited maternally by the sum of the expression when inherited maternally and paternally. RER itself does not include any information about the corresponding locus in other genomes.

### Sequence alignments for SNP-ASE

In parallel to the above method of mapping reads, we also ran the standard, SNP-based allele specific expression pipeline by mapping reads to the B73 AGPv4 reference assembly using a variant-aware aligner HiSat2 trained with a set of known SNPs as described in [44]. The number of reads supporting each parental genotype were used to calculate the proportion of maternal reads for each gene. For comparison across mapping methods, genes were filtered for only those with at least 10 informative reads in both methods. SNP-ASE ratios were calculated for each gene in each direction of the reciprocal cross separately by dividing the number of reads matching the maternal allele by the total number of informative reads. Genes with parent-specific expression were defined as those with a SNP maternal ratio > 0.85 in one direction and < 0.15 in the reciprocal direction.

### Defining imprinting

To define imprinted features using RER, count tables for genes and TEs in each library were loaded into R. For each of the three reciprocal crosses performed in triplicate, DESeq2 [45] was applied using the lfcThreshold = 1 and altHypothesis = "greaterAbs" options to identify features with significant deviations from the 2:1 expected expression difference based on dosage. Each contrast includes features from both parental genomes, so maternal and paternal expression was determined by the direction of the differential expression plus the genome where the feature was annotated. Significant features were further filtered to only strong cases of imprinting where RER was > 0.9 for MEGs and matTEs and < 0.1 for PEGs. To create the final list of imprinted features, maternal features with pericarp-preferred expression were filtered out (see Tissue Dynamics).

### Tissue dynamics

The expression profile of genes and TEs was analyzed for B73 features using previously published analysis [29] using data from [25]. To filter out genes where expression is higher in the pericarp than the endosperm and could thus result in inaccurate imprinting calls [24], expression was compared for 14 dap seeds (the time point used in this study) and 18 dap pericarp. Genes with expression over twice as high in the pericarp over the endosperm were excluded from MEG calls. W22 and PH207 genes corresponding to genes expressed higher in the pericarp were also excluded from MEG calls. No matTEs were identified as potential contaminants using this method. Expression data across all tissues was also used to identify endosperm-preferred expression. Endosperm-preferred expression was defined as genes and TEs where the sum of expression in endosperm and wole seed libraries (26% of libraries) was more than 60% of the sum of expression across all libraries.

### Descriptors

To identify genes that are shared between genome assemblies and annotations, the file gene_model_xref_v4.txt was downloaded from MaizeGDB [26] on 2020/01/22. This file is B73-based and genes with a single corresponding gene in either the pairwise contrast (for venn diagrams) or in both W22 and PH207 (all other analyses) were defined as conserved in maize while remaining genes were defined as variable. This file was also used to define genes that are syntenic with other grasses, with syntenic genes being defined as any gene with a syntenic ortholog in foxtail millet, rice, brachypodium, and sorghum. To identify the nearest gene to each matTE, bedtools closest was used and distances between TE and gene were reported relative to the orientation of the gene.

### RT-PCR

RT-PCR was used to identify cases where TE and gene annotations contribute to a fused imprinted transcript. RNA from B73 x PH207 replicates 1 and 2 along with unsequenced replicate 4 of B73 x W22 was cleaned with the Monarch RNA cleanup kit (T2030L, NEB) and converted into cDNA using the ProtoScript First Strand cDNA synthesis kit using oligo-dT primers (E6300S, NEB). RT-PCR was performed using primers aligning to both the TE and the gene. These sequences were: TGGACATCTTACATTGCTCCAC and CGGGCTTCAAACCAAAAGA for DHH00002Zm00001d02687, GTTGATCTCTGGAACACCAACA and ATGCCCTTGTGCACCTAGTAGT for DHH00002Zm00001d02512, ATACAGCGTGACATTCATTTGC and TATTGAAGTTGGCAGGAAAGGT for RLX11772Zm00001d00001, AATTTCAGTTTCGCCCTATTCA and CCACTGAGCTCCCTCAGTATAAA for RLC00002Zm00001d00679, and AGCTTTCTCCTCCCTTCCTCTA and CGCAGTATTCATCGTCATCATC for DHH00002Zm00001d01432.

### Code availability

Scripts and data files used to process results are available at https://github.com/SNAnderson/Imprinting2020 and https://github.com/kmhiggins/Imprinting_2020. There are no restrictions on code availability.

## Supporting information

S1 Fig Comparison of SNP-ASE and RER for B73 genes accessible using both methods in the B73 x W22 cross. A) The SNP-ASE method applied across both directions of reciprocal crosses, with points colored based on imprinting calls using RER (magenta and blue) or inconsistency across reciprocals using SNP-ASE (green and purple). Colors are consistent across panels. B-C) Comparison of the two SNP-ASE values to RER. Plots are the same as shown in Fig 1B but colored to indicate imprinted genes and genes with genotype-biased expression using SNP-ASE. Genotype bias does not impact RER since the calculation is performed across reciprocals instead of across genotypes. For all panels, values plotted show the average across three biological replicates. Genes are color-coded based on expression pattern, with magenta = MEG, blue = PEG, green = B73 biased, and purple = W22 biased. Genotype bias was defined by SNP-ASE ratios > 0.85 in one direction and < 0.15 in the other direction of reciprocal crosses.
(TIFF)

S2 Fig Distribution of RER for genes and TEs across contrasts. A) Most genes and TEs are expressed near the expected ratio given genome dosage (horizontal line, 0.67), with maternal and paternal expression approaching 1 or 0, respectively. TEs have a more pronounced peak of maternal expression than genes. For TEs, a bimodal peak near 0.67 and 0.33 in contrasts with PH207 suggests that some PH207 TEs may be mapping better to B73 or W22 assemblies due to poor assembly quality of PH207 in intergenic space. For this reason, paternal TEs were not counted in contrasts with PH207 in Fig 3A. B-C) The proportion of parentally biased RER values for different features across contrasts. RER cutoffs for strong maternal and strong paternal are > 0.9 and < 0.1, respectively, and cutoffs for moderate maternal and paternal are > 0.8 or < 0.2, respectively.
(TIFF)

S3 Fig Expression profile of B73 MEGs and matTEs in the endosperm and pericarp using RNA-seq data from Stelpflug et al. Genes with mean expression in the pericarp > 2x the mean expression in the endosperm were filtered from the MEG list due to the potential for seed coat contamination. W22 and PH207 genes corresponding to B73 pericarp-preferred genes were also removed from MEG counts. There were no matTEs filtered out using this method. Heat of each pixel represents the expression value compared to the max in the row.
(TIFF)

S4 Fig RER bias for genes with inconsistent imprinting in Fig 2B. For both MEGs (right) and PEGs (left), the majority of genes do not overlap due to low coverage or have RER values in the same direction as imprinted genes but failed to meet our strict statistical and/or RER threshold. X-axis labels denote the genotype where the gene is imprinted, the type of imprint, and the cross where the imprinting is variable (B = B73, W = W22, P = PH207).
(TIFF)

S5 Fig Expression pattern of imprinted B73 genes and TEs across development using data from Stelpflug et al. Endosperm and seed tissues are shown on the left side of the break, and all other tissues sampled are shown on the right of the break. Endosperm-preferred expression was defined where the sum of the expression across endosperm and seed libraries were more than 60% of the sum of the expression across all libraries. For each plot, endosperm-preferred features are shown above the break and constitutive features are shown below the break. Heat of each pixel represents the expression value compared to the max in the row.
(TIFF)

S6 Fig Presence of PHE1 binding sites within DHH2 family helitrons. Binding motifs from Batista et al. 2019 were identified in all DHH2 helitrons, and the distribution of members with zero, one, or both of the sites were determined. A similar distribution of motifs were found for matTEs and non-imprinted family members, with a lower proportion of elements with at least one motif identified in the not detected set. TEs in the not detected set include TEs that are not expressed and TEs without unique sequence that could be assessed for imprinting.
(TIFF)

S7 Fig IGV views showing examples of reads aligning to matTEs (magenta) near or overlapping MEGs (cyan). Reads are colored by the strand of alignments, where blue = forward strand and red = reverse strand. Green bars show primer positions for the RT-PCR amplifications shown below each example.
(TIFF)

S1 Table Reads and mapping statistics for RNA-seq libraries in this study. (PDF)

S2 Table Gene IDs for conserved imprinted genes plotted in Fig 1C. (PDF)

S3 Table IDs and features of matTEs near MEGs. (PDF)

S1 Data RER output table for B73 and W22 contrast. (XLSX)

S2 Data RER output table for B73 and PH207 contrast. (XLSX)

S3 Data RER output table for W22 and PH207 contrast. (XLSX)

S4 Data Summary table for features of B73 genes and TEs used in this study. (XLSX)
