# Supplementary material for: Widespread imprinting of transposable elements and variable genes in the maize endosperm
Source: PLoS Genet. 2021 Apr 8;17(4):e1009491. doi: 10.1371/journal.pgen.1009491 (PMC8057601; doi:10.1371/journal.pgen.1009491)
Supplement: S1 Table — (PDF) [file pgen.1009491.s008.pdf]

Table S1

Library	Reads	Percent_Unique
BW1	50912503	16.09983
BW2	46955481	16.63385
BW3	43297034	17.88011
WB1	42341423	18.12524
WB2	38350809	14.4479
WB3	53936174	13.86724
BP1	50287029	14.24699
BP2	51260636	16.18052
BP3	45525383	16.54365
PB1	43670832	19.41905
PB2	39706669	18.70737
PB3	41024925	15.71401
WP1	53203456	15.92903
WP2	52838664	16.56664
WP3	41023269	19.63248
PW1	53900552	15.71085
PW2	35267586	18.17527
PW3	35654524	18.32236

Table S1: Reads and mapping statistics for RNA-seq libraries in this study
