# Supplementary material for: Widespread imprinting of transposable elements and variable genes in the maize endosperm
Source: PLoS Genet. 2021 Apr 8;17(4):e1009491. doi: 10.1371/journal.pgen.1009491 (PMC8057601; doi:10.1371/journal.pgen.1009491)
Supplement: S2 Table — (PDF) [file pgen.1009491.s009.pdf]

Table S2

Gene ID	Feature	Genome	Imprinted
GRMZM2G365731 (ARID1)	Zm00001d032832	B73	PEG
AC191534.3_FG003 (VIM104)	Zm00001d019342	B73	PEG
GRMZM2G073700	Zm00001d037209	B73	MEG
GRMZM5G866423 (ARID9)	Zm00001d032096	B73	PEG
GRMZM2G118205 (FIE1)	Zm00001d049608	B73	MEG
GRMZM2G379898	Zm00001d027290	B73	MEG
GRMZM2G365731 (ARID1)	Zm00004b004198	W22	PEG
AC191534.3_FG003 (VIM104)	Zm00004b035225	W22	PEG
GRMZM2G073700	Zm00004b029838	W22	MEG
GRMZM5G866423 (ARID9)	Zm00004b003587	W22	PEG
GRMZM2G118205 (FIE1)	Zm00004b020902	W22	MEG
GRMZM2G379898	Zm00004b000042	W22	MEG
GRMZM2G365731 (ARID1)	Zm00008a004277	PH207	PEG
AC191534.3_FG003 (VIM104)	Zm00008a027204	PH207	PEG
GRMZM2G073700	Zm00008a025023	PH207	MEG
GRMZM5G866423 (ARID9)	Zm00008a003685	PH207	PEG
GRMZM2G118205 (FIE1)	Zm00008a015599	PH207	MEG
GRMZM2G379898	Zm00008a000045	PH207	MEG

Table S2 - Gene IDs for conserved imprinted genes plotted in Figure 1C
