# Supplementary material for: Widespread imprinting of transposable elements and variable genes in the maize endosperm
Source: PLoS Genet. 2021 Apr 8;17(4):e1009491. doi: 10.1371/journal.pgen.1009491 (PMC8057601; doi:10.1371/journal.pgen.1009491)
Supplement: S3 Table — (PDF) [file pgen.1009491.s010.pdf]

Table S3

TE	gene	distance	TE.type	gene.type	order	ratio.BW.gene	ratio.BP.gene	ratio.BW.TE	ratio.BP.TE	gene.variability	gene.expression	TE.expression
DHH00002Zm00001d06247	Zm00001d029042	0	matTE	MEG	DHH	NA	1	NA	1	variable	endo.preferred	endo.preferred
RLG00003Zm00001d01354	Zm00001d031633	-925	matTE	MEG	RLG	1	NA	1	0.841323333	variable	endo.preferred	endo.preferred
RLX19061Zm00001d00001	Zm00001d023985	0	matTE	MEG	RLX	1	1	0.97095182	1	variable	endo.preferred	endo.preferred
RLC00002Zm00001d00679	Zm00001d026623	-797	matTE	MEG	RLC	0.996352427	0.735195542	1	0.701963246	conserved.maize	endo.preferred	endo.preferred
DHH00002Zm00001d01432	Zm00001d004147	0	matTE	MEG	DHH	0.992924908	1	1	1	variable	endo.preferred	endo.preferred
RLC02716Zm00001d00002	Zm00001d005712	-29185	matTE	MEG	RLC	1	1	1	1	variable	endo.preferred	endo.preferred
RLG00003Zm00001d02516	Zm00001d005712	-3087	matTE	MEG	RLG	1	1	1	1	variable	endo.preferred	endo.preferred
RLC00004Zm00001d02983	Zm00001d007488	-852	matTE	MEG	RLC	0.954011986	0.971259533	0.912021829	0.991412204	variable	constitutive	constitutive
RLX11772Zm00001d00001	Zm00001d041755	-1453	matTE	MEG	RLX	1	NA	1	NA	variable	endo.preferred	endo.preferred
DHH00002Zm00001d07691	Zm00001d041887	0	matTE	MEG	DHH	0.982580808	0.846116079	0.986576458	0.961109425	variable	endo.preferred	endo.preferred
RLX11447Zm00001d00001	Zm00001d043716	1670	matTE	MEG	RLX	0.986874555	NA	0.945756767	NA	conserved.maize	endo.preferred	endo.preferred
RLX11446Zm00001d00001	Zm00001d043716	0	matTE	MEG	RLX	0.986874555	NA	0.987866795	0.739333538	conserved.maize	endo.preferred	endo.preferred
DHH00002Zm00001d02512	Zm00001d048646	0	matTE	MEG	DHH	1	NA	1	0.415964714	variable	endo.preferred	endo.preferred
DHH00002Zm00001d02687	Zm00001d050068	0	matTE	MEG	DHH	1	1	1	1	variable	endo.preferred	endo.preferred
DHH00002Zm00001d08890	Zm00001d017481	1078	matTE	MEG	DHH	0.932781433	1	1	0.99597214	variable	endo.preferred	endo.preferred
RLG00017Zm00001dS0065	Zm00001d038034	-2662	matTE	MEG	RLG	0.914617966	0.776546976	0.956875803	0.817167014	variable	constitutive	constitutive
RLG00001Zm00001d12334	Zm00001d046395	-47897	matTE	MEG	RLG	NA	1	1	1	variable	endo.preferred	endo.preferred
RLG00003Zm00001d07748	Zm00001d046395	-3398	matTE	MEG	RLG	NA	1	NA	1	variable	endo.preferred	endo.preferred
RLC00183Zm00001dS0162	Zm00001d046395	-10170	matTE	MEG	RLC	NA	1	NA	1	variable	endo.preferred	endo.preferred

Table S3: IDs and features of matTEs near MEGs
